# Quorum sensing gene *lasR* promotes phage vB_Pae_PLY infection in *Pseudomonas aeruginosa*

**DOI:** 10.1186/s12866-024-03349-7

**Published:** 2024-06-10

**Authors:** Yan Liu, Zhuocheng Yao, Zhenzhi Mao, Miran Tang, Huanchang Chen, Changrui Qian, Weiliang Zeng, Tieli Zhou, Qing Wu

**Affiliations:** Department of Clinical Laboratory, Key Laboratory of Clinical Laboratory Diagnosis and Translational Research of Zhejiang Province, the First Affiliated Hospital of Wenzhou Medical University, Wenzhou, 325000 China

**Keywords:** *Pseudomonas aeruginosa*, Quorum sensing, *lasR*, Phage infection, Phage receptor

## Abstract

**Background:**

Quorum sensing (QS) is a cell density-based intercellular communication system that controls virulence gene expression and biofilm formation. In *Pseudomonas aeruginosa* (*P. aeruginosa*), the LasR system sits at the top of the QS hierarchy and coordinates the expression of a series of important traits. However, the role of *lasR* in phage infection remains unclear. This study aims to investigate the role of *lasR* QS in phage infection.

**Methods:**

The *P. aeruginosa* phage was isolated from sewage, and its biological characteristics and whole genome were analyzed. The adsorption receptor was identified via a phage adsorption assay. Following *lasR* gene knockout, the adsorption rate and bactericidal activity of phage were analyzed. Finally, real-time quantitative polymerase chain reaction (RT-qPCR) was conducted to explore how *lasR* promoting phage infection.

**Results:**

The lytic phage vB_Pae_PLY was isolated and lipopolysaccharide (LPS) was identified as its adsorption receptor. The adsorption rate and bactericidal activity of vB_Pae_PLY were reduced after *lasR* knockout. RT-qPCR results showed that the expression of *galU*, a key gene involved in LPS synthesis, was down-regulated, and several genes related to type IV pili (T4P) were also down-regulated in the *lasR* mutant PaΔ*lasR*.

**Conclusions:**

The study showed that QS *lasR* may promote phage vB_Pae_PLY infection by involving in the synthesis of LPS and T4P. This study provides an example of QS in promoting phage infection and deepens the understanding of phage-bacteria interactions.

**Supplementary Information:**

The online version contains supplementary material available at 10.1186/s12866-024-03349-7.

## Background

*Pseudomonas aeruginosa* (*P. aeruginosa*) is an opportunistic pathogen that uses quorum sensing (QS) signaling molecules to regulate the expression of virulence genes and biofilm [1]. *P. aeruginosa* has a particularly complex QS signaling network, which primarily consists of four interconnected systems (LasI/R, RhlI/R, PQS, and IQS) [2]. In the Las and Rhl systems, the *lasI* and *rhll* genes are responsible for the synthesis of N -(3-oxo-dodecanoyl)- L -homoserine lactone (3- oxo -C12-HSL) and N -(butanoyl)- L -homoserine lactone (C4-HSL), respectively. Then, 3- oxo -C12-HSL and C4-HSL interact with LasR, respectively, to form LasR- 3- oxo -C12-HSL and RhlR- C4-HSL complex to regulate target genes [3]. The PQS system enables the biosynthesis of QS signal molecules 2-heptyl-4-hydroxyquinoline (HHQ) and 2-heptyl-3-hydroxy-4(1 H)-quinolone (PQS, Pseudomonas quinolone signal) [4]. IQS is the fourth and recently discovered QS system, which synthesizes the signaling molecule 2-(2-hydroxyphenyl)-thiazole-4-carbaldehyde (IQS) [1]. Among all the QS systems, the Las tops the signal network. The LasR- 3- oxo -C12-HSL complex positively regulates the expression of receptor and synthase genes in the downstream QS system, thus establishing a regulatory feedforward loop [5]. QS directly or indirectly regulates more than 10% of its genome and about 20% of its bacterial proteome [6, 7], and controls the expression of a series of important traits in *P. aeruginosa* [8, 9].

Phages are viruses that specifically infect bacteria and are abundant in environments associated with their host bacteria. Phages bind to the surfaces of bacterial cells by recognizing adsorption receptors [10]. Several factors relating to the host bacteria affect phage infection, including the surface structure, immune system, growth state, genetic variation, and quorum sensing (QS) [11–13]. Currently, several studies have reported the role of QS in phage infection of host bacteria. QS can regulate phage defense. *P. aeruginosa* used QS to activate the expression of its CRISPR-Cas immune defense system, thereby protecting the bacteria from phage infection [14, 15]. QS also reduced phage infection by down-regulating phage receptors [16, 17]. However, the study by Xuan et al. found that *lasI* QS promoted phage infection in *P. aeruginosa* [18]. In summary, the role of *P. aeruginosa* QS in phage-bacteria interactions is obviously diverse and complex. In the future, more studies are needed to reveal the role of *P. aeruginosa* QS in phage infection.

This research aims to elucidate the role and mechanism of *lasR* QS in phage infection. The study shows that the *lasR* QS may promote phage infection via the biosynthesis of lipopolysaccharide (LPS) and type IV pili(T4P), key components of bacterial surface required for phage adsorption and infection. This finding will help to fully understand the mechanism of phage infection under QS，and guide the application of phage therapy.

## Materials and methods

### Strains, plasmids, and growth conditions

Comprehensive details of the strains and plasmids used in this study are shown in Table S1 in the supplementary materials. All primers used for polymerase chain reaction (PCR) in this study are listed in Table S2. *P. aeruginosa* was cultured in blood plate or Luria-Bertani (LB) broth medium at 37℃. Carbenicillin (Car, 300 µg/mL) and gentamicin (Gen, 200 µg/mL) (Wenzhou Kangtai Biological Technology Co., Zhejiang, China) were added as needed.

### Isolation of the phage

Referring to the methodology outlined in a previous study [19], the phage vB_Pae_PLY was isolated from the sewage of the First Affiliated Hospital of Wenzhou Medical University using PAO1 as the host strain and enriched to reach a titer of 1 × 10^10^ plaque-forming units (PFU)/mL. The purified phage lysate was stored in SM buffer (50 mM Tris-HCl, pH 7.5, 100 mM NaCl, and 8 mM MgSO4) at 4℃ for later use. Then, the double-layer agar plate method was used to determine phage titer. Briefly, 100 µL of 10^9^ colony-forming units (CFU)/mL bacterial host culture was mixed with 100 µL of ten-fold serially diluted phage lysate and incubated for 10 min at room temperature. Subsequently, 8 mL of melted 0.4% soft agar maintained at 45 °C was added to the phage–bacteria mixture. After mixing, the contents of the tube were immediately poured over a pre-prepared confluent monolayer of 1.5% agar. After incubation at 37℃ for 8–12 h, the number of PFUs was calculated. Serially diluted phage suspensions were prepared in triplicate for each dilution factor. The titer of the phage stock (PFU/mL) was calculated as follows:

Titer (PFU/mL) = Number of PFUs × 10 (Volume conversion factor) × Dilution factor.

### Analysis of biological characteristics of the phage

#### Host range determination

Spot assays were performed to determine the host range of the vB_Pae_PLY phage against an additional 40 strains of *P. aeruginosa* clinical isolates. The presence of a clear zone and lysis plaque was recorded as the tested strain being susceptible to the phage [20].

#### One-step growth curve

The one-step phage growth was performed as previously described but with few modifications [21]. The phage vB_Pae_PLY was mixed with the host *P. aeruginosa* PAO1 with a multiplicity of infection (MOI) of 0.01. After adsorption at 37℃ for 10 min, the mixture was centrifuged at 12,000 × *g* for 2 min to remove the unadsorbed phages in the supernatant. The pellet was washed twice with LB broth medium, resuspended in 25 mL of fresh LB broth medium, and incubated with shaking at 37°C, 180 rpm. The samples were then collected at 0, 10, 20, 30, 40, 60, 90, 120 and 240 min, respectively, and the phage titer was determined via the double-layer agar method. The burst size was calculated as the ratio of the final phage titer to the number of initial bacterial cells infected.

#### Transmission Electron Microscopy (TEM)

TEM imaging was performed following a previously established method to observe the morphology of phages [19].

### Whole genome analysis of phage

#### Genomic structural analysis and functional annotation

The genomic DNA of vB_Pae_PLY was extracted using λ phage genomic DNA extraction kit (column type) (Beijing Abigen Biotechnology Co., Ltd., Beijing, China) according to the manufacturer’s instructions. Genome sequencing and analyses were performed at Shanghai Personal Biotechnology Co., Ltd. The genome was annotated using Prokka and eventually corrected manually by using BLASTP to search for similar proteins in the NCBI non-redundant database. The genomic architecture of vB_Pae_PLY was analyzed using Proksee (https://proksee.ca/) and the genome map was visualized [22]. Virulence factors and antibiotic resistance-encoding genes were searched using the Virulence Factor Database (VFDB, http://www.mgc.ac.cn/VFs/main.htm) and the Comprehensive Antibiotic Resistance Database (CARD, https://card.mcmaster.ca/analyze/rgi) webservers, respectively. In addition, phage genome-based life cycle identification was performed using the PhageAI online platform (https://phage.ai/) [19]. The complete genome sequence of vB_Pae_PLY was deposited in the GenBank database under accession number OR689712.

#### Phylogenetic tree analysis

ViPTree (https://www.genome.jp/viptree/) was used to generate viral proteomic tree and classify viruses based on genome-wide similarity. A total of 349 genome sequences of all Pseudomonadota phages of the family Autophagoviridae were selected for the phylogenetic tree analysis by iTOL (https://itol.embl.de/).

### Identification of the phage adsorption receptor

#### Periodate and proteinase K treatments

Bacterial cell surface LPS and outer membrane proteins were destroyed using periodate and proteinase K, respectively [23, 24]. The PAO1 strain was cultured overnight in LB broth medium at 37 °C to an OD_600_ of 1 (approximately 1 ~ 3 × 10^9^ CFU/mL). The bacterial culture was then mixed with 100 mM sodium periodate (NaIO_4_) and incubated at 25 °C in the dark for 2 h. Similarly, a 0.2 mg/mL proteinase K solution (QIAGEN, Germany) was added to the bacterial culture, and the mixture was incubated at 37 °C for 3 h. Following both treatments, the mixture was centrifuged at 12,000 × *g* for 1 min. The cells were washed and re-suspended in 1 mL LB broth medium. A control without NaIO_4_ or proteinase K was included.

The phage adsorption rate was then measured: 10 µL of phage suspension (1 × 10^8^ PFU/mL) was added to 1 mL each of the above bacterial suspensions respectively and incubated at 37 °C with shaking at 180 rpm for 10 min to allow sufficient adsorption of phages. After centrifugation at 12,000 × *g* for 1 min, the phage titer of in the supernatant was measured using the double-layer agar plate method as described above. As a control, the phage titer in the LB broth medium alone was set to 100%. The phage adsorption rate was calculated as follows: Phage adsorption rate (%) = [(Control phage titer - Residual phage titer in supernatant)/ Control phage titer] ×100%.

#### LPS adsorption assay

LPS was isolated from *P. aeruginosa* PAO1 using the LPS Extraction Kit. The LPS adsorption assay was performed with minor modifications, as previously described [18]. Briefly, 50 µL of the extracted LPS was added to 850 µL of LB broth medium, and mixed with 100 µL of phage solution (10^5^ PFU/mL) at 37 °C for 10 min to allow adsorption. For the control sample, 50 µL of phosphate-buffered saline (PBS) was added to the LB broth medium and mixed with the phage. The phage titer in the supernatant and the phage adsorption rate were determined as described above.

### Analysis of the effect of *lasR* on phage infection

#### Construction of the *lasR* knockout mutant (PaΔ*lasR*)

The gene knockout of *lasR* was performed using a previously described method from PAO1 [25]. The plasmids and primers used for the gene knockout are listed in Table S1 and S2, respectively. The *lasR* mutant strain was screened by colony PCR and the target fragment was sequenced (Beijing Luhe Huada Gene Technology Co.,Ltd, China). Finally, transcriptome sequencing technology was used to verify the success of the *lasR* gene knockout.

#### Spot assay

To evaluate phage sensitivity, 3 µL aliquots of 10-fold serial dilutions (10^5^–10^7^ PFU) of each phage were spotted on a bacterial lawn on double-layer agar plates [26]. After incubation at 37 °C for 12 h, plaque formation was observed.

#### Adsorption rate assay

The phage adsorption assay was conducted following a previous methodology with minor modifications [27]. 100 µL of 10^7^ PFU/mL phage solutions were mixed with 900 µL of PAO1 and PaΔ*lasR* bacterial cultures (1 × 10^8^ CFU/mL) separately at an MOI of 0.01, and incubated at 37 °C for 10 min to allow sufficient phage adsorption. The titer of free phage in the supernatant was determined and phage adsorption rates were calculated as described above.

#### Phage-killing experiment

To further compare the effect of the *lasR* gene on phage infection efficiency, time-killing experiments were performed to detect the number of viable bacteria during the phage-killing process with minor modifications based on previous method [19]. Firstly, the PAO1 and PaΔ*lasR* strains were cultured overnight in fresh LB broth medium, and the bacterial suspensions were adjusted to 0.5 McFarland. Then, 30 µL of bacterial suspensions were added to 3 mL of LB broth medium, followed by the addition of 30 µL of phage (1 × 10^9^ PFU/mL). Further, the mixture was incubated at 37 °C with shaking at 180 rpm for 12 h. Following this, 20 µL of the culture was collected at 4, 8, and 12 h intervals, respectively, for viable colony counts.

### Real-Time quantitative PCR (RT-qPCR)

RT-qPCR was performed referring to a previous study [28]. Individual colonies of freshly cultured PAO1 and PaΔ*lasR* strains were grown to the logarithmic phase in the LB broth medium. Then, bacterial RNA was extracted using RNAiso Plus. The total cDNA was synthesized using the PrimeScript™ RT Reagent Kit, and Real-time PCR was performed following the manufacturer’s instructions (Takara Biomedical Technology Co., Ltd.). The levels of mRNA of the target genes were normalized against *rpsL*. Using PAO1 as the control strain, the relative expression was calculated by the 2^−ΔΔCt^ method. All primers used in this study are listed in Table S2.

### Statistical analyses

All the data were expressed as mean ± standard deviation of at least three independent experiments. Statistical significance was determined using an independent two-tailed t-test. The *P*-values of < 0.05, < 0.01, and < 0.001 were denoted by *, **, and ***, respectively, whereas insignificance was denoted by ns. The GraphPad Prism 8.0 software was used for the statistical analysis.

## Results

### Morphology, host range, and one-step growth curve of vB_Pae_PLY

**Fig. 1:**
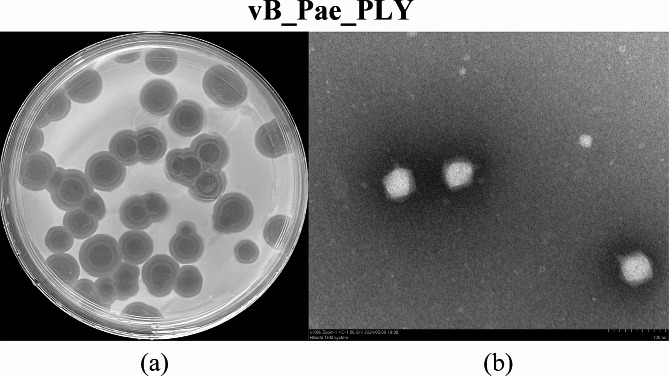
**(a)** Plaque formation on a double-layer agar plate of vB_Pae_PLY; **(b)** Morphology of vB_Pae_PLY under TEM.

**Fig. 2:**
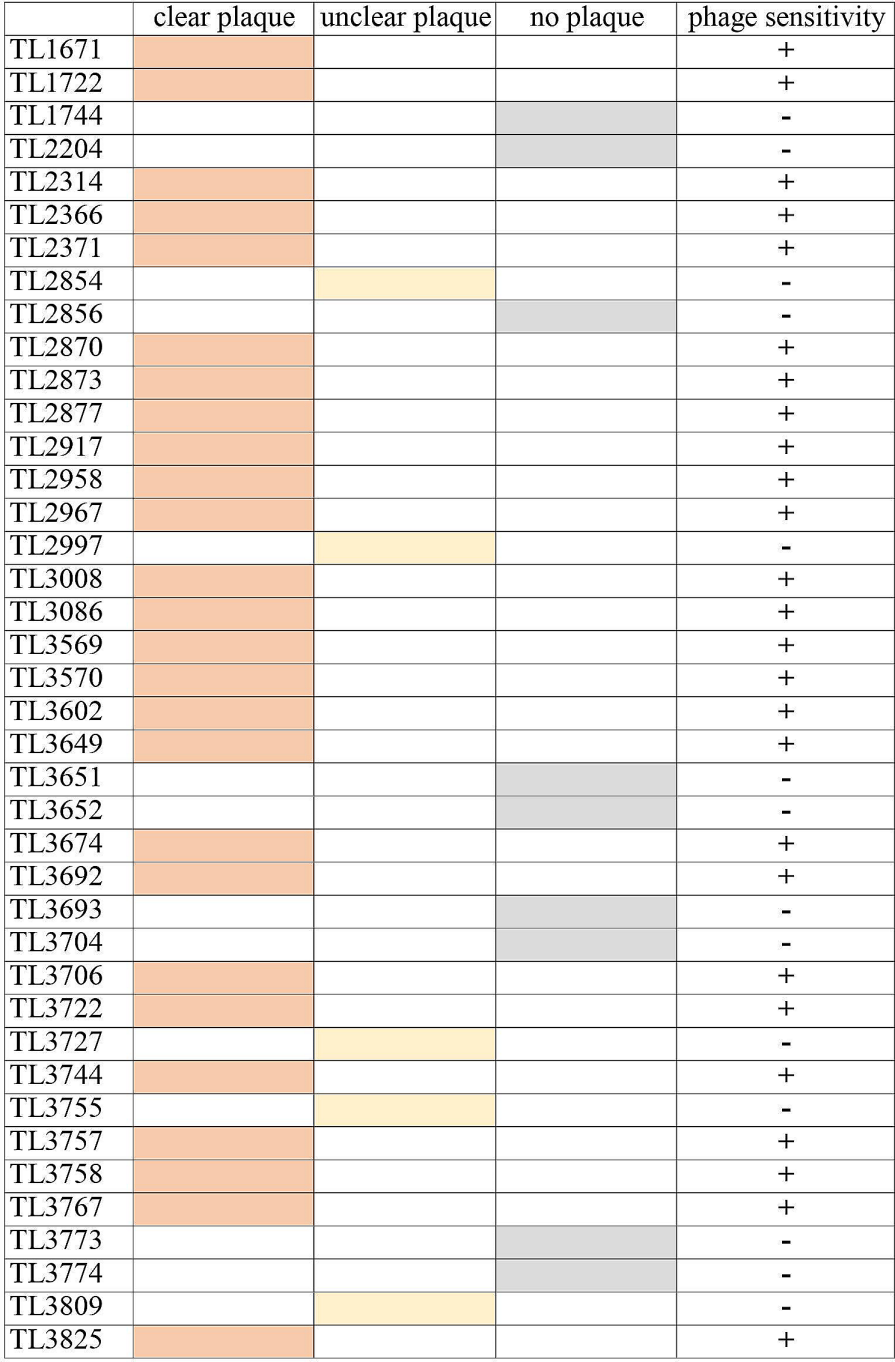
Lytic activity of vB_Pae_PLY against 40 strains of *P. aeruginosa*. +, phage formed a clear zone or plaque; −, phage formed no clear zone nor plaque

Phage plaques with clear morphology and distinct boundaries were obtained on PAO1 (Fig. 1a). TEM observations of the viral particles revealed that vB_Pae_PLY is a short-tailed phage, which had a polyhedral capsid and an almost invisible tail; thus, it was classified to the Autographiviridae family according to the International Committee on Taxonomy of Viruses (ICTV) [29]. The head diameter and tail length of vB_Pae_PLY were approximately 55 and 10 nm, respectively (Fig. 1b). The phage sensitivity analysis of the 40 strains of *P. aeruginosa* showed that vB_Pae_PLY was able to lyse 24 of 40 (60%) clinical isolates to form a clear plaque region on a double-layer agar plate (Fig. 2).

**Fig. 3:**
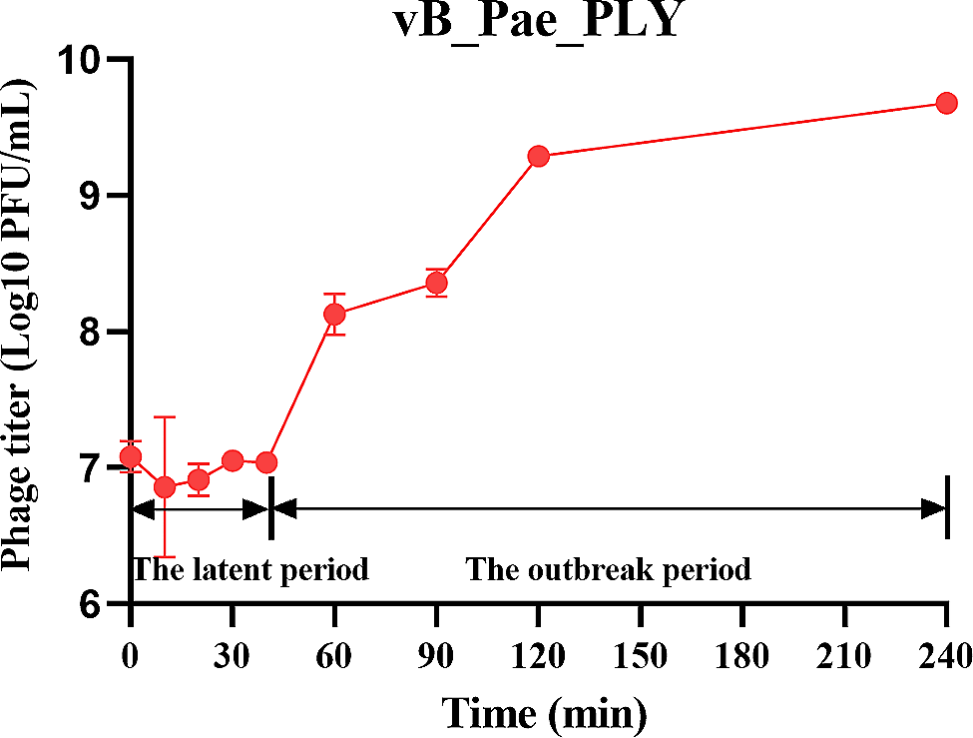
The one-step growth curve showed a latency period of 40 min and high burst size (up to 853 PFU/infected cell)

A one-step growth curve was used to determine the burst size and latent period of viruses. From the one-step growth curve of the phage vB_Pae_PLY (Fig. 3), the latent period, defined as the time between infection and subsequent release of phage virions, was about 40 min, the outbreak period was about 200 min, and the burst size was more than 853 PFU per infected cell.

### Genomic signature of vB_Pae_PLY

**Fig. 4:**
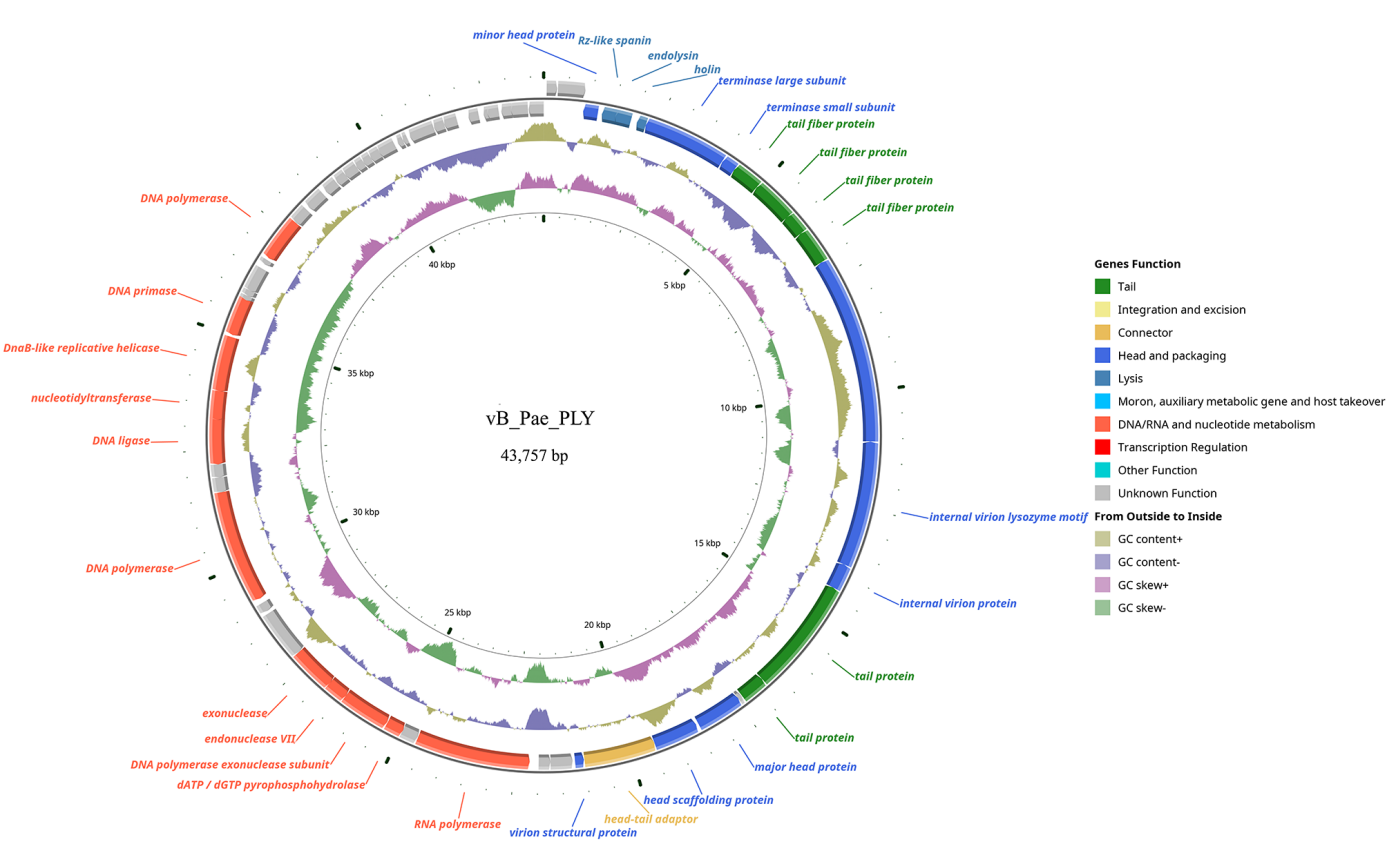
The genomic structure of vB_Pae_PLY. The first circles represent the 64 coding DNA sequences (CDS) of the phage genome. The second circle shows GC content. The third circle shows the GC skew

To better understand the phage vB_Pae_PLY, its genomic DNA was extracted and sequenced. The complete nucleotide sequence of the genome of vB_Pae_PLY has been deposited in the GenBank database (https://www.ncbi.nlm.nih.gov/genbank/) under accession number OR689712. As shown in Fig. 4, vB_Pae_PLY has a dsDNA genome with 43,757 bp and a G + C content of 62.12%. The genome was predicted to contain 64 coding DNA sequences (CDS), which were classified into six functional categories, including “tail protein”, “connector”, “head and packaging”, “lysis”, “DNA/RNA and nucleotide metabolism”, and “hypothetical proteins”. Three CDS encode “lysis” proteins (Rz-like spanin by CDS 4, endolysin by CDS 5, and holin by CDS 6). Among the 64 CDS, 34 encode hypothetical proteins that share similarities with other *P. aeruginosa* phage and have no clear function in the replication and viral infection of vB_Pae_PLY. No potential antibiotic resistance, virulence, or tRNA genes were identified. Information about functional annotation of the 64 CDS and their respective proteins is detailed in Table 1.

**Table 1:** Information about functional annotation of 64 CDS in the vB_Pae_PLY genome

Feature	Location	Product	Function
CDS_3	842–1156	minor head protein	head and packaging
CDS_4	1246–1575	Rz-like spanin	lysis
CDS_5	1533–1877	endolysin	lysis
CDS_6	2012–2209	holin	lysis
CDS_7	2209–4014	terminase large subunit	head and packaging
CDS_8	4023–4328	terminase small subunit	head and packaging
CDS_9	4328–4933	tail fiber protein	tail
CDS_10	4937–5845	tail fiber protein	tail
CDS_11	5838–6296	tail fiber protein	tail
CDS_12	6296–7051	tail fiber protein	tail
CDS_13	7053-11,066	internal virion protein with endolysin domain	head and packaging
CDS_14	11,070 − 13,766	internal virion lysozyme motif	head and packaging
CDS_15	13,766 − 14,311	internal virion protein	head and packaging
CDS_16	14,311 − 16,791	tail protein	tail
CDS_17	16,794 − 17,348	tail protein	tail
CDS_19	17,445 − 18,452	major head protein	head and packaging
CDS_20	18,505 − 19,473	head scaffolding protein	head and packaging
CDS_21	19,477 − 21,009	head-tail adaptor	connector
CDS_22	21,021–21,212	virion structural protein	head and packaging
CDS_25	22,177 − 24,624	RNA polymerase	RNA and nucleotide metabolism
CDS_27	24,976 − 25,347	dATP / dGTP pyrophosphohydrolase	RNA and nucleotide metabolism
CDS_28	25,357 − 26,403	DNA polymerase exonuclease subunit	RNA and nucleotide metabolism
CDS_29	26,400 − 26,840	endonuclease VII	RNA and nucleotide metabolism
CDS_30	26830-27,771	exonuclease	RNA and nucleotide metabolism
CDS_33	29,183 − 31,606	DNA polymerase	RNA and nucleotide metabolism
CDS_36	32,201 − 33,199	DNA ligase	RNA and nucleotide metabolism
CDS_37	33,148 − 33,768	nucleotidyltransferase	RNA and nucleotide metabolism
CDS_38	33,758 − 34,948	DnaB-like replicative helicase	RNA and nucleotide metabolism
CDS_39	34,995 − 35,819	DNA primase	RNA and nucleotide metabolism
CDS_44	36,809 − 37,759	DNA polymerase	RNA and nucleotide metabolism
Another 34 CDS		hypothetical protein	unknown function

### Phage taxonomy and phylogeny analysis

**Fig. 5:**
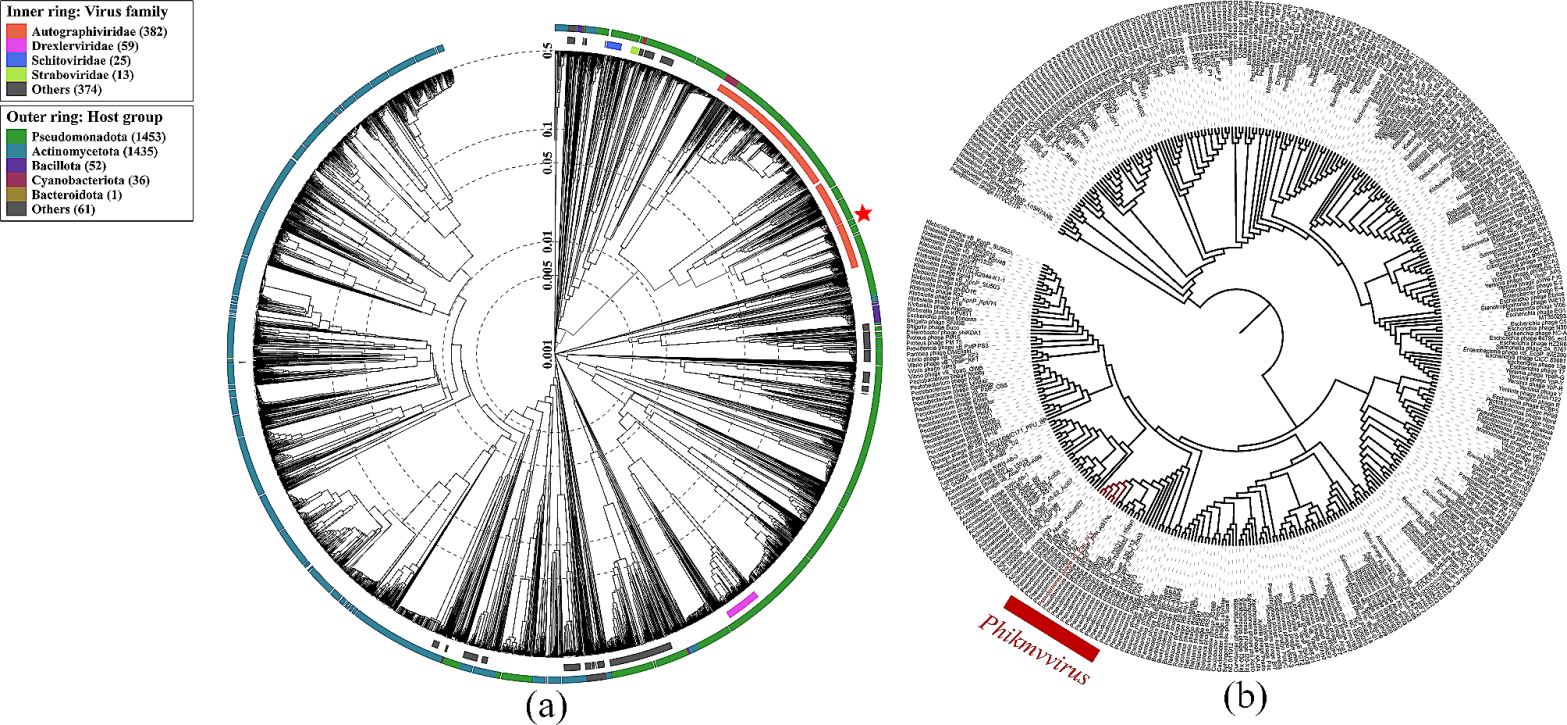
Phage taxonomy and phylogeny analysis. **(a)** Viral proteomic tree; **(b)** Phylogenetic tree based on the 349 genome sequences of Pseudomonadota phages of the family Autophagoviridae

To classify vB_Pae_PLY, a viral proteomics tree was created. The analysis showed that vB_Pae_PLY belonged to the family Autographiviridae, which was consistent with the results observed via TEM (Fig. 5a). A phylogenetic tree based on 349 (including vB_Pae_PLY) genome sequences of all Pseudomonadota phages of the family Autophagoviridae depicted that vB_Pae_PLY was most closely related to the five phages (phage PAXYB1, phage MPK6, phage RLP, phage LUZ19, and phage DL62) of *Phikmvvirus*(Fig. 5b). Therefore, the phage vB_Pae_PLY was assigned to the genus *Phikmvvirus* of the subfamily Krylovinae, the family Autographiviridae, without order classification.

### The adsorption receptor of vB_Pae_PLY

**Fig. 6:**
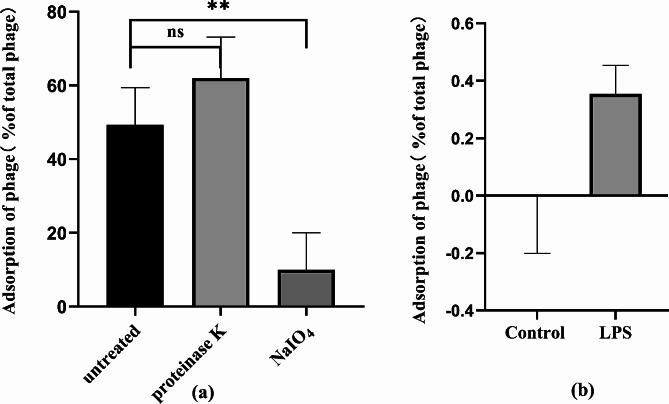
Identification of LPS as an important absorption receptor of vB_Pae_PLY. (**a**) NaIO4 treatment significantly reduced the adsorption of vB_Pae_PLY; (**b**) LPS adsorption assay. The adsorption rate was increased in the LPS-added group compared to that in the control group. **P < 0.01, ns, no significance

Phage adsorption to the bacterial surface is the first and most crucial step in the phage infection process. LPS and outer membrane proteins located on the surface of Gram-negative bacteria can both serve as phage receptors. Proteinase K and NaIO4 were used to disrupt the bacterial surface component to identify the attachment sites of vB_Pae_PLY. PAO1 showed a significant decrease in phage adsorption following NaIO4 treatment (*P <* 0.01), but no observable changes were found after the proteinase K treatment (Fig. 6a). As periodate destroys the structure of LPS and protease K destroys outer membrane proteins, it could be inferred that LPS is an adsorption receptor for vB_Pae_PLY. This result was further confirmed by the LPS adsorption assay, in which phage adsorption to *P. aeruginosa* increased when LPS was added to the reaction system (Fig. 6b).

### The adsorption rate and bactericidal activity of vB_Pae_PLY after *lasR* knockout

**Fig. 7:**
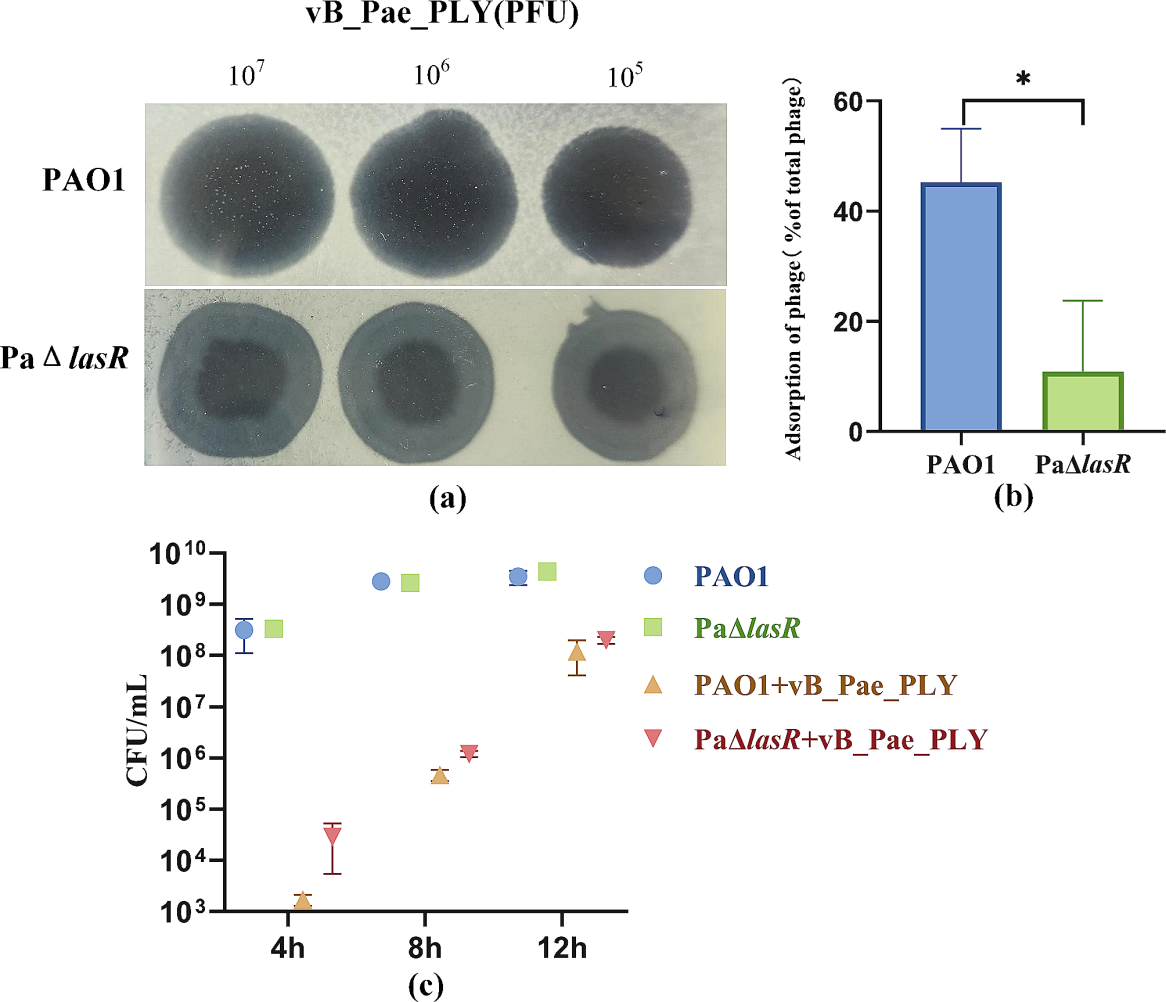
Phage sensitivity assay. (**a**) Ten-fold serial dilutions of the phage vB_Pae_PLY plated on PAO1 and *lasR* mutants PaΔ*lasR*; (**b**) Comparison of adsorption rates of the phage vB_Pae_PLY to PAO1 and PAO1Δ*lasR*; (**c**) Time killing effect in the presence or absence of phage at an MOI of 10. *P < 0.05

Transcriptome analysis showed that *lasR* expression decreased significantly, and QS was down-regulated, indicating that *lasR* was successfully knocked out (Figure S1). The study further investigated the influence of the Las QS system on phage infection. The results of the spot assay showed that *lasR* deletion reduced the clarity of clear patches formed by vB_Pae_PLY (Fig. 7a). The adsorption rates of phages were compared, which revealed that PaΔ*lasR* showed a significant decrease in phage adsorption compared to wild-type PAO1 (*P* < 0.05) (Fig. 7b). The results suggested that *lasR* knockout may influence phage sensitivity by reducing phage adsorption to *P. aeruginosa*.

Additionally, colony-counting results showed that compared to wild-type PAO1, the bactericidal effect of vB_Pae_PLY against PaΔ*lasR* was reduced (Fig. 7c). At 4 h and 8 h, the colony count in the PaΔ*lasR* group was higher than that in the wild group, and the phage resistant bacteria load of PaΔ*lasR* was higher than that in the wild group. At 12 h, there was little difference in bacterial load between the two groups. These results showed that *lasR* QS inhibition weakened the bactericidal efficiency of the phage vB_Pae_PLY.

### The expression of LPS and T4P synthesis genes after *lasR* knockout

**Fig. 8:**
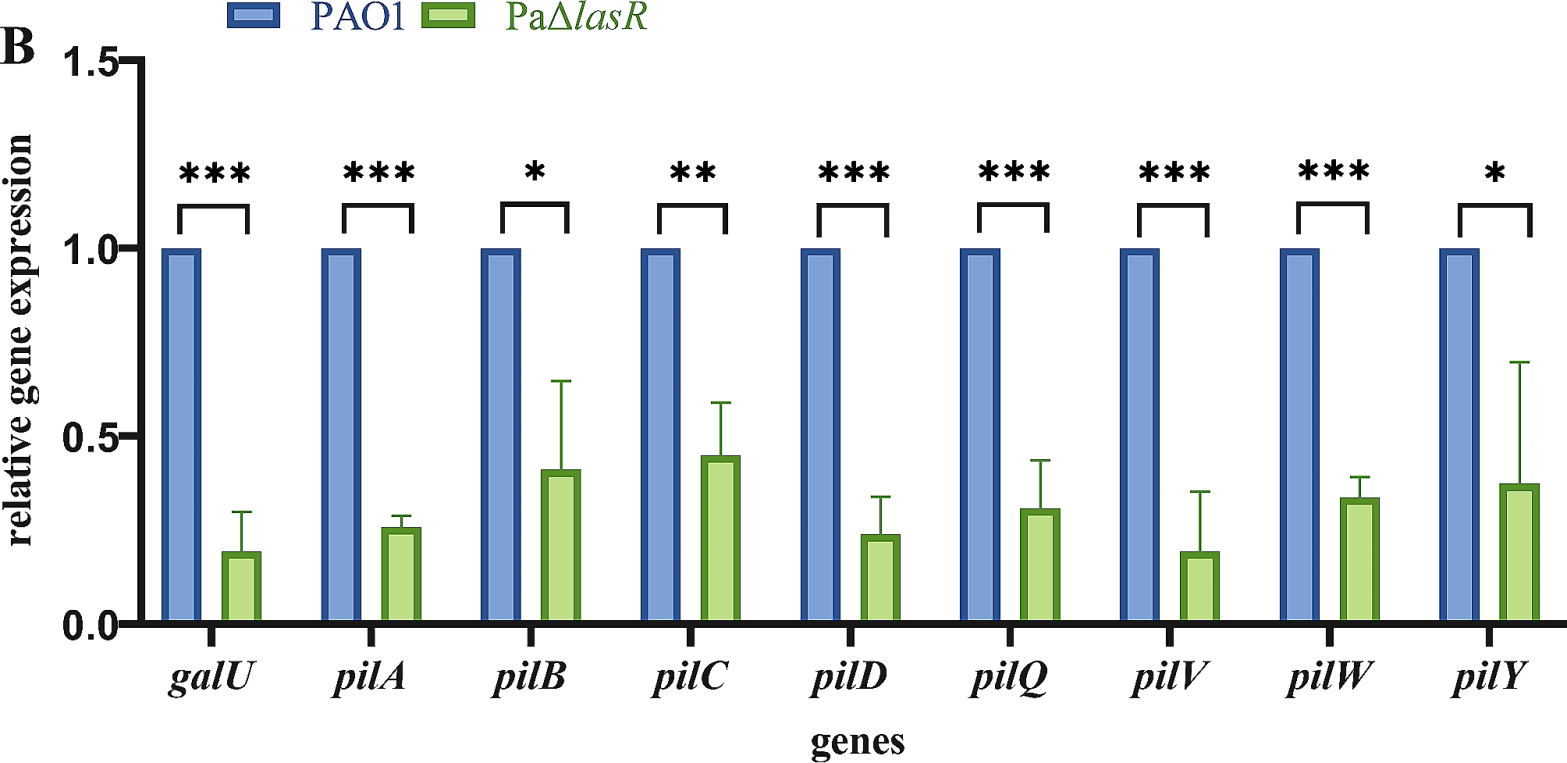
*lasR* knockout down-regulated the expression of phage absorption-related genes. The reference gene was *rpsL*. *P < 0.05, **P < 0.01, and ***P < 0.001

A differential gene expression experimen was performed by RT-qPCR to gain insights into the mechanism of changes in the sensitivity of PaΔ*lasR* to the phage. The expression level of LPS synthesis gene *galU* was significantly decreased after *lasR* knockout. In addition, T4P synthesis related genes (including *pilA*, *pilB*, *pilC* and *pilD*, *pilQ* and *pilV*, *pilW* and *pilY*) were also markedly down-regulated (Fig. 8**)**. As LPS is a key adsorption receptor for phages and T4P is important in phage adsorption, the down-regulation of LPS and T4P related genes caused by *lasR* knockout may be the main reason for the decreased phage sensitivity.

## Discussion

Studies have shown that in some Gram-negative bacteria like *Vibrio* and *Escherichia coli* (*E. coli*), QS regulated phage resistance by down-regulating phage receptors on the cell surface. M Mozammel et al. indicated that QS may mediate phage resistance by reducing or eliminating O antigen expression in *Vibrio cholerae* [30]. Down-regulation of the outer membrane protein K (OmpK) receptor mediated by QS in *Vibrio anguillarum* reduced phage adsorption [17]. In *E. coli*, the QS system reduced phage receptors [16, 31]. Additionally, the study by Xuan *et a*l. revealed that N-acyl homoserine lactones (AHLs) protected *Shewanella baltica* from phage infection by decreasing LPS-mediated phage adsorption [32]. The above studies all hypothesized that QS negatively regulates the expression of receptors required for phage infection. However, while the phenomena and mechanisms of QS-controlled phage resistance have been observed in these bacteria, it has not been clear whether QS has similar effects in other bacteria. Interestingly, the present study found that the knockout of *lasR* in *P. aeruginosa* PAO1 decreased the phage sensitivity and adsorption (Fig. 7), indicating that *lasR* positively regulated phage infection. This is consistent with previous observations in *P. aeruginosa* [18, 33]. This study demonstrated that *lasR* promotes phage infection in *P. aeruginosa*.

Phage adsorption to the bacterial surface is the first and most critical step in phage infection [34, 35]. Molecules on the bacterial surface, such as LPS, pili, peptidoglycan components, and outer membrane proteins, can all be attachment sites for phage tails [35–38]. LPS is related to virulence in various Gram-negative bacteria, and is also the receptor of many phages [39–42]. Some LPS-specific phages against *P. aeruginosa*, and phage-resistant strains generated by mutations in LPS biosynthetic genes have been described in the literature [43–47]. T4P plays a central role in the expression of many phenotypes including motility, multicellular behavior, sensitivity to phages, natural genetic transformation, and adhesion [48, 49]. Studies show that many *P. aeruginosa* phages utilize pili as the primary receptor to infect cells, and mutations or inhibition of T4P synthesis may reduce the adsorption [18, 40, 41, 46, 47]. Downregulation of *pilA*, *pilB* and *pilQ* genes reduced T4P-mediated phage adsorption to protect *P. aeruginosa* from phage infection [41, 50].

RT-qPCR analysis in the present study revealed that *galU* and T4P-related genes (*pilA*, *pilB*, *pilC* and *pilD*, *pilQ* and *pilV*, *pilW* and *pilY*) were significantly decreased in the PaΔ*lasR* (Fig. 8). LPS is composed of O antigen, core oligosaccharide, and lipid A. *galU *encodes UDP-glucose pyrophosphorylase (GalU), *and* was found to be involved in the synthesis of the core region of *P. aeruginosa* LPS [43, 51]. The current study demonstrates that the phage vB_Pae_PLY utilizes surface LPS as the receptor for adsorption (Fig. 6). Thus, the reduced phage sensitivity in PaΔ*lasR* can be largely attributed to the impaired LPS synthesis. In addition, pili also play an important role in phage infection; therefore, downregulation of T4P may also be another reason for reduced phage sensitivity. LPS and T4P are important virulence factors for the opportunistic pathogen *P. aeruginosa*. On the other hand, serving as phage receptors, LPS and T4P facilitate phage infection with their synthesis being positively regulated by QS. Therefore, *lasR* QS inhibition will affect the synthesis of bacterial LPS and T4P, resulting in reduced phage adsorption with LPS and T4P as receptors, and weaken the bactericidal effect of phages to a certain extent.

## Conclusions

In conclusion, this study demonstrates that *lasR* may promote phage infection by positively regulating the biosynthesis of LPS and T4P. Disrupting *lasR* expression leads to decreased phage sensitivity. Given the complex and multifaceted role of QS in host-phage interactions, the future research is needed to uncover the various mechanisms by which QS participates in phage infection.

### Electronic supplementary material

Below is the link to the electronic supplementary material.

Supplementary Material 1

Supplementary Material 2

Supplementary Material 3

## Data Availability

The complete genome sequence of phage vB_Pae_PLY presented in this study is openly available in [GenBank, OR689712].

## Abbreviations

*Pseudomonas aeruginosa*: *P. aeruginosa*
QS: Quorum sensing
RT-qPCR: Real-Time quantitative polymerase chain reaction
LPS: Lipopolysaccharide
T4P: Type IV pili-related
AHL: N-acylhomoserine lactones
3-oxo-C12-HSL: N -(3-oxo-dodecanoyl)- L -homoserine lactone
C4-HSL: N -(butanoyl)- L -homoserine lactone
HHQ: 2-heptyl-4-hydroxyquinoline
PQS: 2-heptyl-3-hydroxy-4(1 H)-quinolone
IQS: 2-(2-hydroxyphenyl)-thiazole-4-carbaldehyde
LB: Luria-Bertani
Car: Carbenicillin
Gen: Gentamicin
PFU: Plaque-forming unit
CFU: Colony-forming unit
VFDB: Virulence Factor Database
CARD: Comprehensive Antibiotic Resistance Database
PCR: Polymerase chain reaction
PBS: Phosphate-buffered saline
MOI: Multiplicity of infection
TEM: Transmission electron microscopy
CDS: Coding DNA sequences
ICTV: International Committee on Taxonomy of Viruses
OmpK: Outer membrane protein K
GalU: UDP-glucose pyrophosphorylase
