# Supplementary material for: Quorum sensing gene lasR promotes phage vB_Pae_PLY infection in Pseudomonas aeruginosa
Source: BMC Microbiol. 2024 Jun 10;24:207. doi: 10.1186/s12866-024-03349-7 (PMC11163716; doi:10.1186/s12866-024-03349-7)
Supplement: Supplementary file 1 — Supplementary Material 1 [file 12866_2024_3349_MOESM1_ESM.docx]

| **Strain/plasmid** | **Characteristic** | **Source** |
| --- | --- | --- |
| ***E. coli* strain** |  |  |
| DH5α | Cloning strain | Solarbio |
| DH5α/pUCP-Red | DH5α carrying pUCP-Red Plasmid | Lab stock |
| ***P. aeruginosa* strain** |  |  |
| PAO1 | Wild type strain | Lab stock |
| PAO1/pUCP-Red | PAO1 with pUCP-Red plasmid, Car^R^ | Lab stock |
| PAO1Δ*lasR* | PAO1 mutant with *lasR* knocked out, Gm^R^ | This study |
| **Plasmid** |  |  |
| pJQ200SK | Gene knockout vector, Car^R^ | Lab stock |
| pUCP -Red | PUCP plasmid carrying red recombinant gene  （containing *exo*, *bet*, *gam* genes regulated by arabinose promoter）; Car^R^ | Lab stock |

**Table S1 Strains and plasmids used in this study**

Car^R^, carbenicillin-resistant; Gm^R^, gentamicin-resistant.
