# Supplementary material for: Quorum sensing gene lasR promotes phage vB_Pae_PLY infection in Pseudomonas aeruginosa
Source: BMC Microbiol. 2024 Jun 10;24:207. doi: 10.1186/s12866-024-03349-7 (PMC11163716; doi:10.1186/s12866-024-03349-7)
Supplement: Supplementary file 2 — Supplementary Material 2 [file 12866_2024_3349_MOESM2_ESM.docx]

| **Primers** | **Sequence (5'-3')** | **Product size(bp)** |
| --- | --- | --- |
| **Primers for gene knockout** | | |
| *Gm^R^*-F | GCTGCTGCCCAAGGTTGC | 835 bp (gentamicin resistance gene) |
| *Gm^R^*-R | CCGATCTCGGCTTGAACGA |  |
| *lasR* knockout up-F | CGTCGCCGAACTGGAAAAG | 528 bp |
| *lasR* knockout up-R | GCAACCTTGGGCAGCAGCGCCATAGCGCTACGTTCTTC |  |
| *lasR* knockout down-F | TCGTTCAAGCCGAGATCGGATGGCCGTTAATTTGGGTCT | 505 bp |
| *lasR* knockout down-R | GCGCTCCTTGAACACTTGAG |  |
|  | **Primers for** **qPCR** |  |
| qPCR*-lasR*-F | ATGCTCAAGGACTACGCACTGC | 207 bp |
| qPCR*-lasR*-R | CACACCGAACTTCCGCCGAAT |  |
| qPCR-*rpsL*-F | TTACACGACCGCCACGGATCA | 155 bp |
| qPCR*-rpsL-R* | CGTATACACCACCACGCCGAAA |  |
| qPCR-*galU*-F | GACGACCTGTGCCTGAACCT | 127 bp |
| qPCR-*galU*-R | CGCCGTACTTGTTGGTCTCTTC |  |
| qPCR-*pilA*-F | CTGTTGAAGAGTCGCTGTCG | 118 bp |
| qPCR-*pilA*-R | ACACCCAACTTGTTGGCATC |  |
| qPCR-*pilB*-F | GACAAGTCCACCCAAGAGGA | 129 bp |
| qPCR-*pilB*-R | CTTCTCGTAGGGCTCGAAGT |  |
| qPCR-*pilC*-F | GGTCGTTCCGCAGTTCCAATCC | 108 bp |
| qPCR-*pilC*-R | GCAAGCCACCACTCCTGTAGGA |  |
| qPCR-*pilD*-F | TGAGCCTGTGGTCGGTGTTCTG | 130 bp |
| qPCR-*pilD*-R | AGGATGGTCAGCGGCAGGATCT |  |
| qPCR-*pilQ*-F | TGAGCCTGTGGTCGGTGTTCTG | 105 bp |
| qPCR-*pilQ*-R | AGGATGGTCAGCGGCAGGATCT |  |
| qPCR*-pilV*-F | GCCACGCAATCCGACTTCTTCA | 118 bp |
| qPCR*-pilV*-R | TCACCTGTTCCGCCCAGCAT |  |
| qPCR*-pilW*-F | GCTTCGTCGAACAGGCCAACAT | 180 bp  103 bp |
| qPCR*-pilW*-R  qPCR*-pilY*-F  qPCR*-pilY*-R | TCTGGTAGAGGCGGTCCTTGTC  GCCGACGCTGCCTACTACTATG  TCTGCCTTTGCTCTGTGCTCAC |  |

**Table S2 Primers used in this study**
