# Supplementary material for: Quorum sensing gene lasR promotes phage vB_Pae_PLY infection in Pseudomonas aeruginosa
Source: BMC Microbiol. 2024 Jun 10;24:207. doi: 10.1186/s12866-024-03349-7 (PMC11163716; doi:10.1186/s12866-024-03349-7)
Supplement: Supplementary file 3 — Supplementary Material 3 [file 12866_2024_3349_MOESM3_ESM.docx]

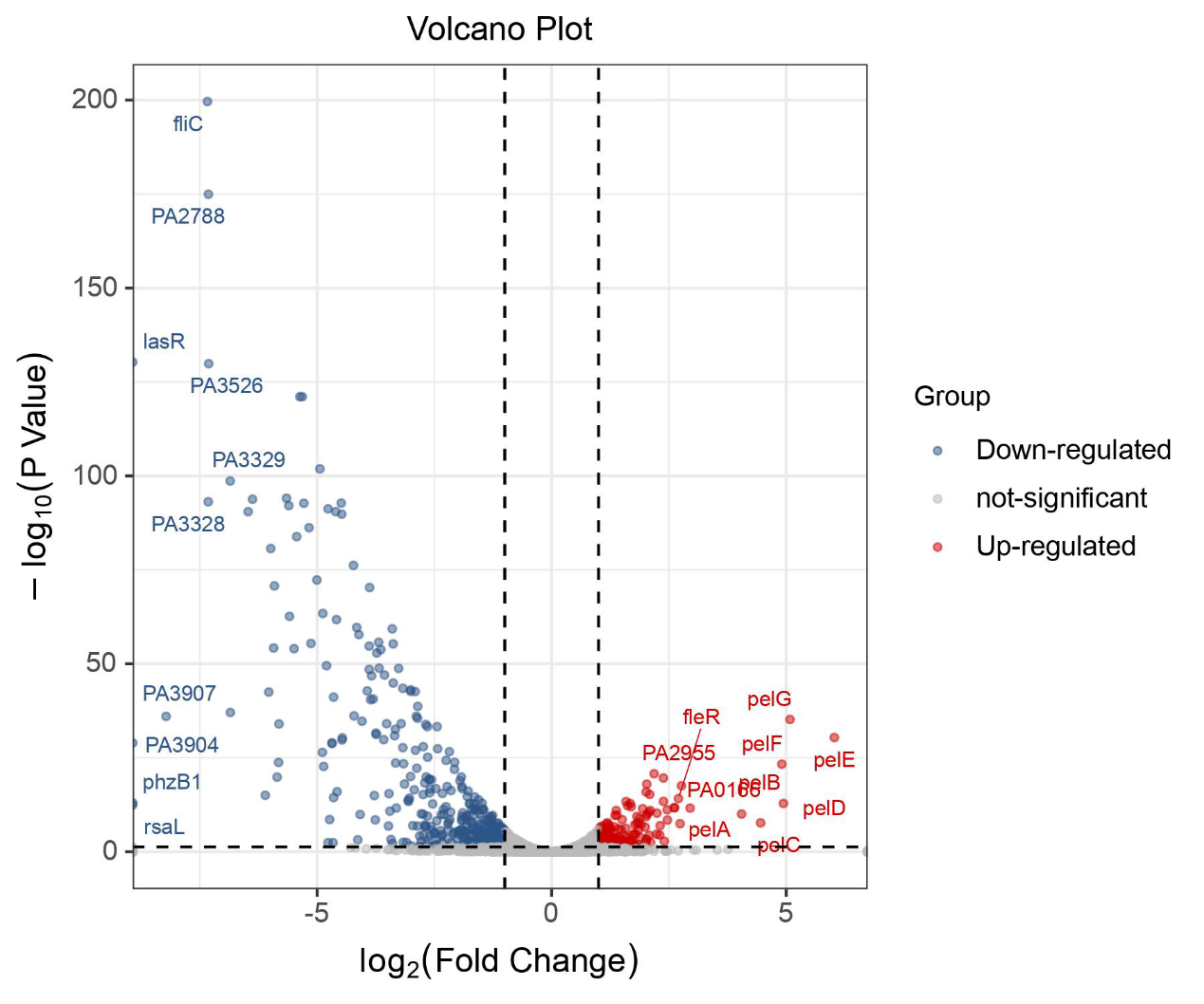


**Figure S1. A volcano plot showing the distribution of the differentially expressed genes after *lasR* knockout. Numerous genes were downregulated (represented by blue dots).**
